# The effect of short-form video addiction on undergraduates’ academic procrastination: a moderated mediation model

**DOI:** 10.3389/fpsyg.2023.1298361

**Published:** 2023-12-15

**Authors:** Jin Xie, Xinyu Xu, Yamei Zhang, Yuxin Tan, Dazhou Wu, Mingjian Shi, Hai Huang

**Affiliations:** ^1^Education Research Institute, China University of Geosciences (Wuhan), Wuhan, China; ^2^School of Mechanical Engineering and Electric Information, China University of Geosciences (Wuhan), Wuhan, China; ^3^School of Psychology, Central China Normal University, Wuhan, China; ^4^School of Psychology, Beijing Normal University, Beijing, China; ^5^Political Officer Education Department, Dalian Naval Academy, Dalian, China

**Keywords:** short-form video addiction, academic procrastination, attentional control, boredom proneness, college students

## Abstract

**Background:**

Short-form videos have become one of the most popular ways for people to entertain and relax. However, the intense interest in short-form videos has given rise to short-video addiction, which poses risks to both physical and mental health of individuals. Undergraduates are one of the important users for short-form videos, and the influence of short-form video addiction calls for more attention. This study aimed to investigate the association between short-form video addiction and academic procrastination among undergraduates, exploring the role of executive functions (i.e., attentional control) and personality traits (i.e., boredom proneness) in the association.

**Methods:**

Using stratified random cluster sampling method, the data of 1,047 college students were used in the study. All variables were measured by empirical instruments, and all instruments were highly reliable. Mediation and moderation analysis was conducted using Model 4 and 7 in PROCESS macro powered by SPSS.

**Results:**

Results revealed that short-form video addiction not only directly impacted academic procrastination but also placed indirect effect on academic procrastination through attentional control. Furthermore, the mediating effect of attentional control was contingent upon individuals’ boredom proneness. Higher levels of boredom proneness weakened the impact of short-form video addiction on attentional control.

**Conclusion:**

The findings expand our knowledge of the negative effects of short-form video addiction and the underlying mechanisms, providing implications for mitigating undergraduates’ academic procrastination.

## 1 Introduction

The rapid development of technology and the Internet has transformed people’s lives, and short-form video applications (apps) such as TikTok have swept across the world. Short-form videos are typically less than 15 min in length, mostly between 1 and 5 min, with concise content and a clear theme (Yang et al., 2022). According to GWI (2023), TikTok has attracted 4 billion global downloads from January 2018 to November 2022, becoming the social media platform with the highest download numbers (Chen et al., 2020). As the pioneer of short-videos, Chinese short-form video apps (e.g., Douyin, Kwai) have massive domestic impact. Until 2022, users of short-form videos have reached 962 million, which included 91.5% of the overall Internet users (China Internet Network Information Center, 2022). However, the high impact of short-form videos brought both considerable advantages and potential risks (Chen et al., 2020). It has been found that the overuse of the Internet and other various Internet-based applications (such as online gaming and social networking services) poses a real threat to individuals’ mental health, interpersonal relationships, and quality of life. Corresponding to the phenomenon, broad Internet addiction has gradually attracted attention from the mass media and researchers (Marin et al., 2021). Yet, the impact of specific Internet addictions, including short-form video addiction (SFVA), grant a closer examination (Chen et al., 2021).

Due to the technological design, short-form videos are prone to excessive use and addiction, especially for students and adolescents (Wang et al., 2023). For content creators, the short-form video platforms offer a variety of autonomous functions, including taking, editing, and posting short-form videos. Although content of the videos can be highly varied, most videos are intended to be entertaining for more followers and “likes” to satisfy the emotional need of content creators (Zhang et al., 2019). For audiences, the short-form video apps are often designed to continue playing related videos before manually stopping, which could keep the individuals watching for a long time. As a relatively novel type of mental health problems, short-form video addiction poses a new risk to people’s mental health and social life. Physically, short-form video addiction can cause poor vision, decreased physical fitness, and possibly chronic diseases and other disorders (Liu et al., 2021). In terms of mental health, it can result in emotion regulation problems (Liu et al., 2021; Ye et al., 2022), reduced feelings of well-being (Miron et al., 2019; Ye et al., 2022), and even problematic behaviors such as suicide (Miron et al., 2019; Chalermchutidej et al., 2023). Furthermore, among students, it has been found that short-form video addiction can also lead to poor sleep quality and academic burnout (Chalermchutidej et al., 2023; Vansoeterstede et al., 2023).

Academic procrastination is one of the negative constructs that could be detrimental to academic success. To be specific, academic procrastination is defined as the act of failing to complete academic tasks that should be done properly, often accompanied by emotions such as stress and anxiety (Caroline et al., 2010). However, academic procrastination is a common issue for college students, with some studies report incidence of academic procrastination as high as 70% (Cheng and Xie, 2021; Ma et al., 2022). Continuous academic procrastination not only hinders students’ academic achievement (Bytamar et al., 2020), but also prevents them from effectively regulating emotions and experiencing fulfillment (Balkis, 2013). Studies have shown the relationship between various forms of internet addiction and academic procrastination (Hayat et al., 2020), yet little is known about the impact of short-form video addiction on academic procrastination. Based on the existing evidence, we aimed to examine the direct and indirect effect (attentional control) of short-form video addiction on academic procrastination and explore how personality traits (boredom proneness) might affect this relationship.

### 1.1 Short-form video addiction and academic procrastination

Since short-form video addiction is a type of specific internet addiction, short-form video addition might have similar effects as internet addiction on academic procrastination (Aznar-Diaz et al., 2020). According to the temporal motivation theory, people tend to prefer tasks that offer quicker rewards when time is limited, and postpone those with more distant rewards (Steel, 2007). Using analytical algorithms of massive user database, short-form video apps can provide personalized and engaging content (Yeh et al., 2017; Krivonogova et al., 2022; Hongying and Christian, 2023). The student audience can experience immediate satisfaction and entertainment rewards in a relatively short amount of time (Yeh et al., 2017). Thus, short-form videos afford students to procrastinate in high-demanding tasks, such as academic assignment and self-regulated learning (Miyake and Kane, 2022).

Moreover, short-form video addiction has been shown to be positively associated with various mental health problems, such as symptoms of depression, anxiety, and stress (Sabir et al., 2020). These mental health problems have been identified as significant factors contributing to academic procrastination (Steel, 2007). Meanwhile, previous studies have revealed that short-form videos addiction can diminish people’s motivation to learn (Ye et al., 2022). Overusing short-form videos can foster a short-term oriented mindset, which seeks immediate pleasure and satisfaction. This mindset may diminish expectations for future academic achievements because academic performance typically demands long-term effort and dedication (Albursan et al., 2022). Students might perceive that they cannot attain quick academic rewards, leading to a loss of confidence in their studies and they cannot complete their academic tasks on time. Thus, individuals with short-form video addiction are prone to academic procrastination. Thus, the following hypothesis is put forward:

*H1*: short-form video addiction has a positive effect on academic procrastination.

### 1.2 The mediating role of attentional control

Compared to other specific internet addictions, considering the design and traits of short-form video, individuals with short-form video addiction are vulnerable to cognitive impairment, which could negatively impact their academic outcomes. Hence, attentional control emerged as a distinctive mediator. Attentional control, a cognitive function that regulates the allocation of attention, is significantly linked to executive control (Derryberry and Reed, 2002; Hopfinger and Slotnick, 2020). Derryberry and Reed (2002) proposed that effective attentional control requires not only the active engagement of cognitive resources to focus on relevant information but also the suppression of irrelevant information, which highlights the importance of both promoting goal-directed behavior and restraining interference (Derryberry and Reed, 2002).

The ability to control their attention might be impaired by short-form video addiction. Studies have found that exposure to television shows, characterized by high arousal and quick change to focus, could impair capacity to maintain concentration on other tasks (Swing et al., 2010). Highly arousing content typically elicits strong excitement in the brain, leading to an increased allocation of cognitive resources for information processing and a more frequent shift in attentional focus (Christakis et al., 2004). Consequently, the cognitive resources are directed toward visual and emotional stimuli, rather than being utilized for task-related cognitive functions. This diversion of resources diminishes the allocation of attention to the task at hand. In comparison to television, short-form videos are inherently as arousing with faster pace of information exchange (Ophir et al., 2009; Moisala et al., 2016; Peng et al., 2018), which could result in higher degrees of dysfunction in attentional control.

Attentional control is essential for on-time academic completion. Students with high attentional control are more adaptive to distractions and external stimuli, leading to reduced attentional bias to unrelated information in performing academic tasks (Ma et al., 2022). Alternatively, students with impaired attentional control might spend more time on distractions such as short-form videos, even if they intend to concentrate in academic tasks. From a neurological standpoint, the fMRI study by Dong et al. (2015) have shown that individuals with internet gaming disorders are in a state of imbalance between executive control and the reward network, and a decrease in executive control leads to an increase in motivation-seeking and craving. With the imbalanced systems, individuals are more likely to prioritize short-term satisfaction for reduced cravings and lose sight on long-term outcomes (Brand et al., 2019). Although the imbalance between executive control and reward network was not yet found in individuals with short-form video addition, we speculate that the potential impairment on attentional control would also suffer from poor executive functions. Therefore, the individuals would prioritize short-term cravings (watching short-form videos) compared to long-term success (academic achievement), resulting in academic procrastination. Our hypothesis is presented as follows:

*H2*: Attentional control mediates the relationship between short-form video addiction and academic procrastination.

### 1.3 The moderating effect of boredom proneness

Researchers have found individual differences in the effect of various internet addiction on individual outcomes (Zhang et al., 2019). Neuroticism, among personality traits, have received more attention than others. In the effect of short-form video addiction on attentional control, boredom proneness might play a significant role. Boredom proneness is a personality trait that encompasses emotions such as boredom, unhappiness, restlessness, a lack of enthusiasm, and a sense of meaninglessness (van Tilburg et al., 2013). According to Eastwood et al. (2012), boredom proneness can be defined as an aversive state of wanting to, but being unable to, engage in satisfied activities (Eastwood et al., 2012). Individuals with high and low boredom proneness might receive different impact on their executive functions from short-term video addiction, whereas the specific difference have not yet been investigated. From practical and theoretical perspective, opposing hypotheses were formed regarding the potential moderation.

On the one hand, short-form video addicted student who are high in boredom proneness might suffer from higher level of impairment in attentional control. Boredom proneness has been investigated as an indicator to various internet addiction, including broad internet addiction (Chou et al., 2018), smartphone addiction (Yang et al., 2020), social media addiction (Malik et al., 2023). Individuals high in boredom proneness are also more likely to have problems related to low attentional control, including low sustained attention and increased symptoms of ADHD (Malkovsky et al., 2012). Thus, compared to college students with low boredom proneness, those with high boredom proneness might be predisposed to low attentional control, leading to higher vulnerability to short-form video proneness.

On the other hand, boredom proneness might counteract the impact of short-form video addiction on attentional control. Boredom can drive individuals to escape from monotonous environment built by short-form video apps. Due to the design of short-form video apps, short-form videos are played continuously, and the followed videos are personalized based on user data. Thus, the short-form video apps create an “echo chamber” experience for the users, pushing forward more centralized content with similar information and viewpoints. According to the boredom driven decision-making model, boredom motivates individuals to avoid and find ways to overcome situations that lack sufficient information or stimulation (Seiler et al., 2022). After frequent usage of the apps, even if the content of short-formed videos is arousing and entertaining, people with high boredom proneness might be less engaged with the monotonous environment (Yuan and Wang, 2022). As a result, boredom proneness might serve as a protecting factor for the deleterious effect of monotony and long-term sensory deprivation on executive functions caused by short-form video addiction (Kimberly, 1998; Das et al., 2016; Li and Jia, 2022). Since the past literature does not provide a clear direction of moderating effect of boredom proneness, the current study proposes the following hypothesis:

*H3*: Boredom proneness moderates the relationship between short-form video addiction and attentional control.

### 1.4 The present study

**Figure 1:**
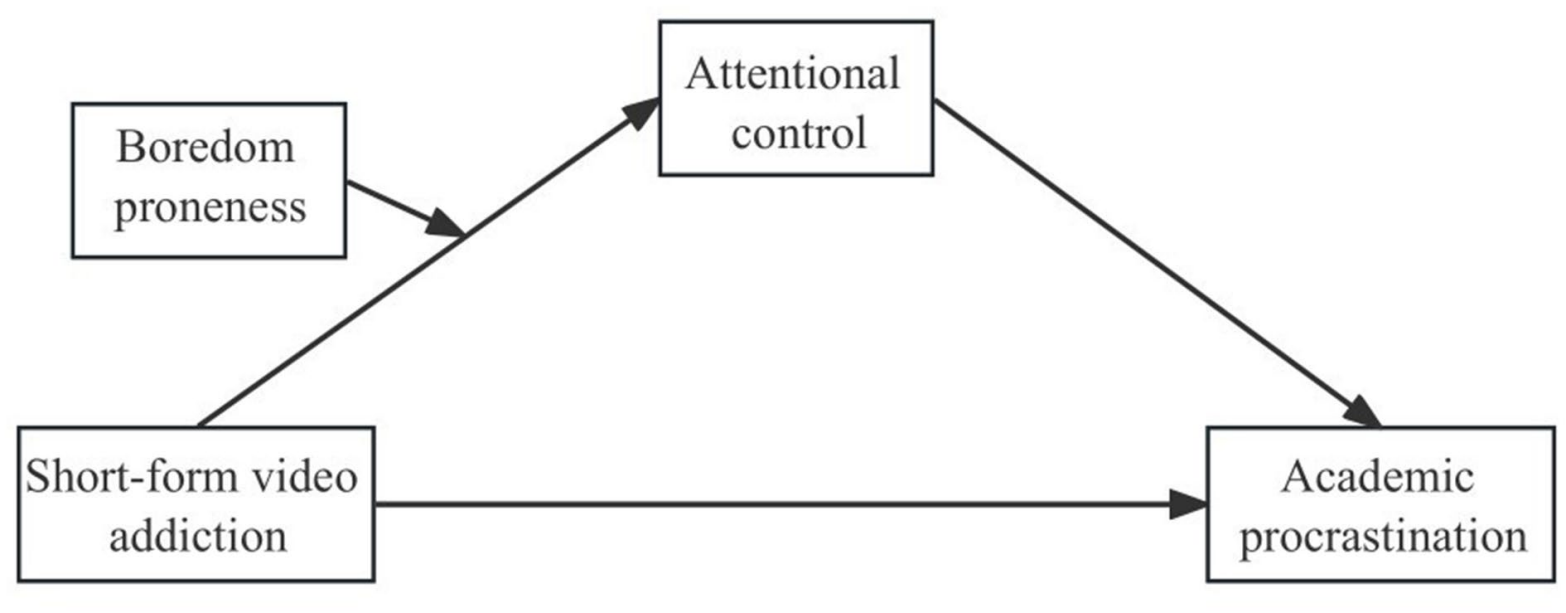
Conceptual model of research.

In conclusion, the current study proposes a moderated mediation model, presented in Figure 1. We hypothesize that short-form video addiction would place direct effect on academic procrastination in college students. We then hypothesize that the effect would be mediated be attentional control. Finally, the effect of short-form video addiction on attentional control is hypothesized to be moderated by boredom proneness.

## 2 Methods

### 2.1 Participants and procedure

Stratified random cluster sampling method was used to determine the survey sample. Researchers will randomly select two classes at each grade level for the distribution of the questionnaires. A total of 1,176 questionnaires were distributed to students in grades 1–3 from seven universities in central China. Local lecturers distributed hyperlinks or QR codes of the questionnaire to university students on WeChat for them to fill in. After the student entered the main page of the survey, an online informed consent form was displayed. If the student did not disagree with the survey objective in the informed consent form, he/she clicked “Next” to start the survey. The student could stop the survey if he/she objected to it. This Internet-based online questionnaire survey was completely anonymous, voluntary, and non-commercial. After deleting the missing data questionnaires, there were 1,047 valid questionnaires with an effective recovery rate of 89.03%. The participants’ ages ranged from 17 to 25 years old (M_age_ = 20.08, SD = 1.34). There were 593 boys and 454 girls. Among the students, 310 (29.6%) were freshmen, 246 (23.5%) were sophomores, and 491 (46.9%) were juniors.

### 2.2 Measurements

#### 2.2.1 Short-form video addiction

Short-form video addiction was assessed using the self-developed Short-Form Video Addiction Scale (SFVAS), which was adapted from the Internet Addiction Diagnostic Questionnaire (IADQ) (Young, 1999). This scale was translated into the Chinese version and was verified to have good validity and reliability. Specifically, two changes were made to the IADQ. (1) the term “internet/online” was changed to “short-form video”; (2) the scale was changed to a 5-point Likert scale ranging from 1 (not at all) to 5 (always). It included items such as “You always spend more time on short-form video apps than you originally planned.” Higher scores indicate more severe short-form video addiction. In this study, Cronbach’s alpha was 0.872, with good reliability.

#### 2.2.2 Attentional control

Attentional control was measured by the Attentional Control Scale (ACS), which includes 8 items that form two dimensions of attentional focus (e.g., “I have difficulty concentrating when music is playing in the room”) and attentional shift (e.g., “I am slow to switch from one task to another) (Carriere et al., 2013). Participants rated each item on a 5-point scale from 1 (none at all) to 5 (always). A higher total score indicates higher attentional control. In this study, Cronbach’s alpha of attentional focus and attentional shift were 0.808 and 0.770 respectively, suggesting good reliability.

#### 2.2.3 Boredom proneness

Boredom proneness was assessed using the Boredom Proneness Scale (BPS) for College Students, which includes 30 items that form two dimensions of external and internal stimuli (Shihua et al., 2010). An example item is “I can be patient.” Participants rated each item on a 5-point scale from 1 (strongly disagree) to 5 (strongly agree). A higher total score indicates higher boredom proneness. In this study, Cronbach’s alpha of external stimuli and internal stimuli were 0.891 and 0.825 respectively, suggesting good reliability.

#### 2.2.4 Academic procrastination

Academic procrastination was measured by the Academic Procrastination Scale (APS) developed by Solomon and Rothblum (1984), which includes 18 items (e.g., Do you procrastinate on this task?). Participants rated each item on a 5-point scale from 1 (none at all) to 5 (always). A higher total score indicates higher academic procrastination. The Cronbach’s alpha was 0.863.

### 2.3 Data analysis

Descriptive statistics and Pearson correlation analysis were also conducted using SPSS 20.0. Then, a hierarchical regression analysis was performed to examine the main effects of the independent variables on the dependent variable. The hypothesized mediating role of attentional control and moderating effect of boredom proneness were examined using the PROCESS macro in SPSS (Model 4 and Model 7). All continuous variables were standardized, and the interaction terms were computed from these standardized scores. The bias-corrected bootstrapping method generates 95% confidence intervals for these effects from 5,000 resamples of the data. 52 The 95% confidence interval excluding zero indicates a significant effect.

## 3 Results

### 3.1 Test of common method bias

The result of Harman’s single factor test showed the first common factor explained 15.79% (less than 40%) of the variance of all items, indicating that common method bias was not a problem.

### 3.2 Descriptive statistics

Means and standard deviations, and Pearson correlations among the variables, are presented in Table 1. Short-form video addiction was significantly negatively correlated with attentional control (*r* = −0.23, *p* < 0.001), and significantly positively correlated with boredom proneness (*r* = 0.34, *p* < 0.001) and academic procrastination (*r* = 0.17, *p* < 0.001); attentional control was significantly negatively correlated with boredom proneness (*r* = −0.35, *p* < 0.001) and academic procrastination (*r* = −0.37, *p* < 0.001); boredom proneness was significantly positively correlated with academic procrastination (*r* = 0.32, *p* < 0.001).

**Table 1:** The descriptive statistics and Pearson correlations.

	*M*	SD	1	2	3	4
1 Short-form video addiction	1.816	0.701	–			
2 Attentional control	3.114	0.669	−0.23^***^	–		
3 Boredom proneness	3.266	0.766	0.34^***^	−0.35^***^	–	
4 Academic procrastination	1.827	0.409	0.17^***^	−0.37^***^	0.32^***^	–

^*^p < 0.05, ^**^p < 0.01, ^***^p < 0.001.

### 3.3 Test of the mediation model

**Figure 2:**
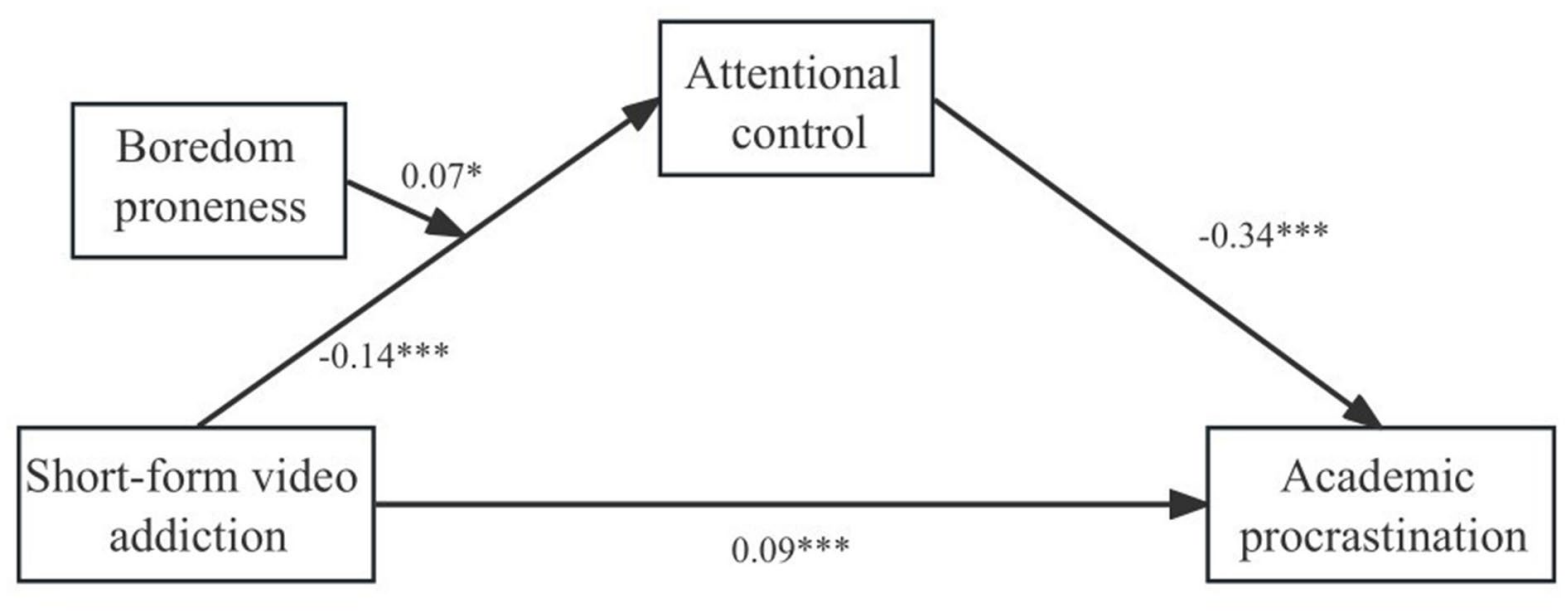
The path coefficients in the moderated mediation model. ^*^p < 0.05, ^***^p < 0.001.

The study variables were first standardized. The results of mediation model are presented in Table 2 and Figure 2. Short-form video addiction significantly positively predicted academic procrastination (*β* = 0.17, *p* < 0.001). Short-form video addiction significantly negatively predicted attentional control (*β* = −0.23, *p* < 0.001). When short-form video addiction and attentional control were tested together as predictors of academic procrastination, short-form video addiction still significantly positively predicted academic procrastination (*β* = 0.09, *p* < 0.001), while attentional control negatively predicted it (*β* = −0.34, *p* < 0.01).

**Table 2:** The mediating effect of attentional control.

Outcome	Predictors	*β*	*SE*	*p*	*R^2^*	*F*
AP	Gender	0.08	0.06	<0.01	0.053	14.691^***^
	Age	0.07	0.04	0.16		
	SFVA	0.17	0.03	<0.001		
AC	Gender	−0.01	0.06	0.75	0.057	15.666***
	Age	0.01	0.04	0.77		
	SFVA	−0.23	0.03	<0.001		
AP	Gender	0.08	0.06	<0.01	0.164	40.956^***^
	Age	0.07	0.03	0.11		
	AC	−0.34	0.03	<0.001		
	SFVA	0.09	0.03	<0.001		

SFVA, Short-form video addiction; AC, Attentional control; AP, Academic procrastination.

### 3.4 Tests of the moderated mediation

**Figure 3:**
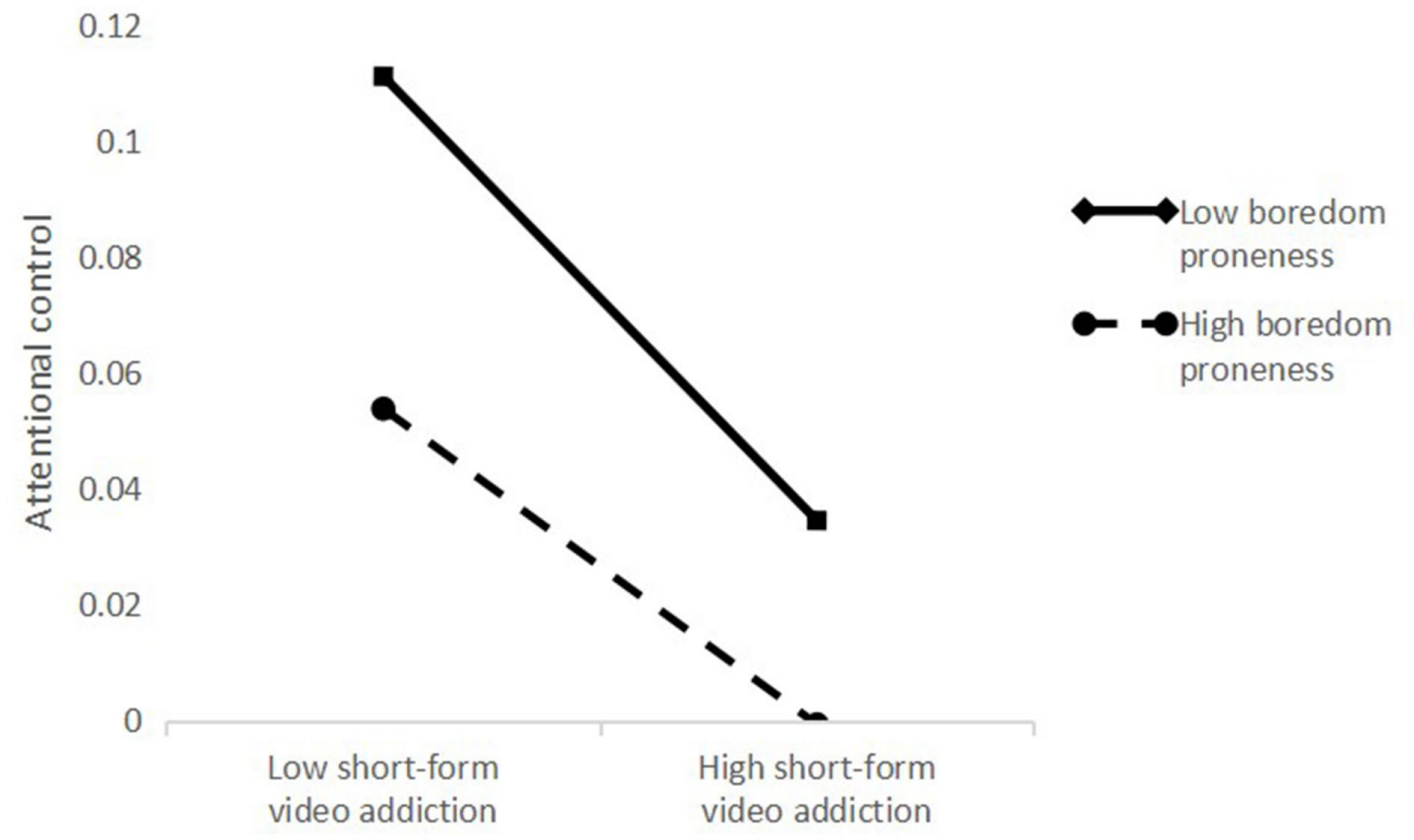
Moderating effect of boredom proneness on short-form video addiction and attentional control.

The study variables were first standardized. Table 3 shows the results of the tests of moderated mediation. Boredom proneness was tested as a moderator of the association between short-form video addiction and attentional control. Short-form video addiction, boredom proneness, and their interaction significantly predicted attentional control (*β* = −0.14, *p* < 0.001; *β* = −0.32, *p* < 0.001; *β* = 0.07, *p* < 0.05). To interpret the moderating role of boredom proneness, simple slopes analysis was conducted using the Johnson-Neyman method (Hayes and Preacher, 2014), as presented in Figure3. The effect of short-form video addiction on attentional control was significant under a range of boredom proneness scores. For college students with lower boredom proneness, the effect of short-form video addiction on attentional control was stronger.

**Table 3:** The moderating effect of boredom proneness.

Outcome	Predictors	*β*	*SE*	*p*	*R^2^*	*F*
Attentional control	Gender	0.20	0.06	0.74	0.153	31.302^***^
	Age	0.02	0.03	0.96		
	SFVA	−0.14	0.32	<0.001		
	BP	−0.32	0.03	<0.001		
	SFVA×BP	0.07	0.03	<0.05		

SFVA, Short-form video addiction; AC, Attentional control; BP, Boredom proneness; AP, Academic procrastination.

## 4 Discussion

The present study examined the association between short-form video addiction and academic procrastination, and the mediating and moderating effect on the association. Results showed that short-form video addiction was positively associated with academic procrastination. Attentional control was found to mediate the association, meaning that short-form video addiction lowered college students’ attentional control, thereby contributing to higher levels of academic procrastination. Additionally, boredom proneness as a personality trait moderated the relationship between short-form video addiction and attentional control.

### 4.1 The relationship between short-form video addiction and academic procrastination

It was found that short-form video addiction is positively associated with academic procrastination, confirming our hypothesis H1. First, short-form videos are typical “small pleasure” as they are entertainment that is considered to take up only a small amount of time. Based on Silver and Sabini (1981) procrastination theory, individuals unconsciously spend significant time on “small pleasures,” thereby occupying the time meant for daily work tasks and leading to task procrastination (Silver and Sabini, 1981). For students suffering from short-form videos addictions, short-form videos are carriers of such “small pleasures.” People with short-form videos addiction may spend more time browsing short-form videos for entertainment and continuously checking for news and messages from their friends (Ryan et al., 2014). They give in to pleasant short-term temptations (such as browsing short-form videos) which can provide instant gratification (Nong et al., 2023). Meanwhile, they often postpone the tasks (such as writing a term paper) which are perceived as stressful, frustrating, or boring (Pychyl et al., 2000). Therefore, students who overuse short-form videos often indulge in short-form videos that provide short-term pleasure and delay the aversive academic tasks (Adrian et al., 2016), leading to academic procrastination.

### 4.2 The mediating role of attentional control

In accordance with our hypothesis H2, results of the current study also discovered that attentional control partially mediated how short-form video addiction affects academic procrastination. Adding the mediation effect to the model significantly elevated the model fit. Higher levels of short-form video additions are accompanied by higher level of exposed information with sustained concentration, which maximizes the user’s attention capacity. Short-form videos are typically presented in a continuous and rapidly switching format, with an immediate sense of satisfaction (Peng et al., 2018). This instant pleasure can stimulate the brain’s reward center, leading to a reinforcement cycle. Students crave more immediate gratification, which can interfere with their long-term attention control (Prabu and Jung-Hyun, 2015). More importantly, although the results are consistent with previous studies that investigated attentional control with overuse of other technologies (e.g., television), there are reasons to believe that short-form videos might have caused more damage to children’s attentional control, compared to television or broad internet addition (Woods and Woods, 2016). Traditional TV programs are well-produced and long videos that lasted over 10 min with information of consistent themes, while the production of short-form videos is inconsistent with diverse styles and themes. Thus, in comparison, short-form videos are much more attention-grabbing and attention-shifting (Woods and Woods, 2016), which could lead to more severe cognitive impairment for addictive users.

With impaired attentional control caused by short-form video addiction, college students were more likely to engage in academic procrastination. When individuals encounter unrelated stimuli (such as social media, short-form videos, message notifications), they struggle to control impulsive actions and start working towards their goals (Cardoso-Leite and Bavelier, 2014). Moreover, individuals with difficulties in attentional control often experience negative emotions such as anxiety and depression when they find it challenging to resist temptations and complete tasks (Adrian et al., 2016). This emotional burden not only has adverse effects on mental health but also makes it harder for individuals to focus on tasks, further preventing them from performing tasks that require high cognitive resources and attention (Rebetez et al., 2018).

### 4.3 The moderating effect of boredom proneness

The study revealed that boredom proneness plays a moderating role in the relationship between short-form video addiction and attentional control, thereby influencing the level of academic procrastination. The result is consistent with our hypothesis H3. Interestingly, although boredom proneness itself was negatively associated with attentional control, the interaction between boredom proneness and short-form video addiction was positively associated with attentional control. The positive association indicated that compared to college students with low boredom proneness, those with high boredom proneness are less impacted by short-form video addiction regarding their attentional control.

According to the sensation-seeking theory, individuals who are more prone to boredom tend to seek novel and exciting experiences (Hunter et al., 2016). When college students who are more prone to boredom develop short-form video addiction, they may actively seek out more diverse and thrilling content (by searching for other videos) to avoid the monotonous environment. Thus, their experience of watching short-form videos might be more controlled by themselves and not dictated by the “echo chamber.” This may mitigate the negative impact of short-form video addiction on their attentional control. Conversely, students with low boredom proneness are more likely to trapped in the echo chamber, continuously shifting their attention between short-form videos, which could further impair their attentional control.

Furthermore, college students with high boredom proneness might have a more profound experience with mental health benefits that could protect them from impaired attentional control. For college students with high boredom proneness, watching short-form videos brought positive emotional experiences, expanding their cognitive resources and cognitive ability and serving as a protective factor from short-video addiction (Pychyl et al., 2000). People who are prone to boredom frequently have significant feelings of loneliness and low self-esteem (Farmer and Sundberg, 1986; Vodanovich, 2003). According to the Interaction of Person-Affect-Cognition-Execution (I-PACE) model, internet addiction has compensatory effect when faced with the threat to relationship and the need for self-existence (Williams, 2009; Brand et al., 2019). Similarly, watching short-form videos compensated for the emotional needs for bored college students, leading to positive emotional experiences and increased self-esteem. According to the broaden-and-build theory of positive emotions, positive emotions can enhance cognitive resources and improve attentional performance (Fredrickson, 2001). Thus, for students who are easily bored, their executive functions are not as impacted by the excessive use of short-form videos.

## 5 Implications and limitations

The study findings have important implications for research and practical applications. First, since short-form video addiction is relatively new and still developing, previous literature has not yet examined how short-form video addiction played a part in academic procrastination. Our study aims to address this research gap by providing a theoretically driven model that focuses on cognitive processes and individual differences. Practically, results of the study may be helpful to disseminate information among college students about the adverse effects of short-form video addiction to reduce its negative impacts. Besides, considering the mediating role of attentional control between short-form video addiction and academic procrastination, educators can guide students using mindfulness to enhance their attentional control, thus reducing academic procrastination (MacDonald and Olsen, 2020). Moreover, students with low boredom proneness are addicted to short-form videos are more at risk for cognitive impairment. Due to the recency of short-form video addiction, specific treatment options are not available. Using broad internet addiction as an analog, cognitive behavioral therapy (CBT), acceptance and commitment therapy (ACT), and medication might be effective options (Winkler et al., 2013). Meanwhile, it is necessary to adopt a dialectical perspective when considering boredom proneness. On the one hand, based on the negative association between boredom proneness and other variables, universities staff should be mindful of students who are high in boredom proneness. On the other hand, because boredom proneness may mitigate the negative effects of excessive short-form video use on attentional control, schools and parents do not need to overtly worry about students feeling bored.

There are several limitations that should be acknowledged in this study. First, due to the use of cross-sectional data, we cannot establish causal relationships. The cross-sectional data also prevents us to explore the bi-directional effect between short-form video addiction and academic procrastination. Past studies found that academic procrastination is a predictor to internet addiction (Tra and Gken, 2020), which could be extended to short-form video addiction. Future research should consider employing experimental or longitudinal designs to address causality and bi-directional effects. Additionally, the study assumed linear relationship between short-form video addiction and attentional control. The moderating effect of boredom proneness could also be the result of the non-linear relationship between the two variables with diminishing returns in extreme value.

Furthermore, the study sample might not be representative of the college student population, as our sample was based on convenience sampling (Iiker et al., 2015). Also, our study only included college students. Past research on short-form video addiction have focused on teenagers or high schoolers, whose attentional control is still developing. Thus, it is possible that the impact of short-form video addiction is more pronounced among teenagers. Future studies should aim to recruit participants from different age groups to better understand the potential age-related differences.

In light of the recent surge of short-form video usage and short-form video addiction, future studies also should investigate in more mediating variables on the effect between short-form video addiction and academic procrastination. For example, time perspective and time perception might be an important mediating variable for understanding the impact of short-form video addiction on individual’s psychological processes. Since short-form videos are short in duration, watching short-form videos may create a false perception of time (Krivonogova et al., 2022). Therefore, college students might spend a large amount of time watching short-form videos without accurate awareness of scheduling, leading to academic procrastination. Also, the academic outcomes of the current study might not be comprehensive. Future studies should also include other outcome variables of academic adjustment other than academic procrastination (Clinciu and Cazan, 2014; Wang et al., 2021).

## 6 Conclusion

The results of this study yielded the following conclusions. (1) Short-form video addiction had a significant positive predictive effect on academic procrastination. (2) Short-form video addiction not only directly predicted academic procrastination among college students but also indirectly affected it via attentional control, which partially mediated the relationship. (3) Boredom proneness moderated the relationship between video addiction and attentional control. When the level of boredom proneness was high, the negative predictive effect of short-form video addiction on attentional control was diminished. Moreover, in terms of the main effect, boredom proneness had a negative effect on attentional control.

## Data availability statement

The original contributions presented in the study are included in the article/supplementary material, further inquiries can be directed to the corresponding author.

## Ethics statement

The studies involving humans were approved by Ethics Committee of China University of Geosciences (Wuhan)-Research Center for Psychological Science and Health. The studies were conducted in accordance with the local legislation and institutional requirements. Written informed consent for participation in this study was provided by the participants' legal guardians/next of kin.

## Author contributions

JX: Writing – original draft, Writing – review & editing. XX: Writing – review & editing, Data curation, Methodology, Validation, Investigation, Funding acquisition. YZ: Writing – original draft.YT: Writing – review & editing. DW: Writing – review & editing. MS: Writing – review & editing. HH: Writing – original draft, Writing – review & editing.
